# The *Phytophthora infestans* Haustorium Is a Site for Secretion of Diverse Classes of Infection-Associated Proteins

**DOI:** 10.1128/mBio.01216-18

**Published:** 2018-08-28

**Authors:** Shumei Wang, Lydia Welsh, Peter Thorpe, Stephen C. Whisson, Petra C. Boevink, Paul R. J. Birch

**Affiliations:** a Division of Plant Sciences, University of Dundee (at The James Hutton Institute), Invergowrie, Dundee, United Kingdom; b Cell and Molecular Sciences, The James Hutton Institute, Invergowrie, Dundee, United Kingdom; University of California, Berkeley

**Keywords:** crop disease, filamentous plant pathogen, pathogenicity, secretome, virulence

## Abstract

The oomycete potato blight pathogen Phytophthora infestans secretes a diverse set of proteins to manipulate host plant immunity. However, there is limited knowledge about how and where they are secreted during infection. Here we used the endoplasmic reticulum (ER)-to-Golgi secretion pathway inhibitor brefeldin A (BFA) in combination with liquid chromatography-electrospray tandem mass spectrometry (LC-MS/MS) to identify extracellular proteins from P. infestans that were conventionally secreted from *in vitro*-cultured hyphae. We identified 19 proteins with predicted signal peptides that potentially influence plant interactions for which secretion was attenuated by BFA. In addition to inhibition by the apoplastic effector EPIC1, a cysteine protease inhibitor, we show that secretion of the cell wall-degrading pectinesterase enzyme PE1 and the microbe-associated molecular pattern (MAMP)-like elicitin INF4 was inhibited by BFA *in vitro* and *in planta*, demonstrating that these proteins are secreted by the conventional, Golgi-mediated pathway. For comparison, secretion of a cytoplasmic RXLR (Arg-[any amino acid]-Leu-Arg) effector, Pi22926, was not inhibited by BFA. During infection, whereas INF4 accumulated outside the plant cell, RXLR effector Pi22926 entered the plant cell and accumulated in the nucleus. The P. infestans effectors, the PE1 enzyme, and INF4 were all secreted from haustoria, pathogen structures that penetrate the plant cell wall to form an intimate interaction with the host plasma membrane. Our findings show the haustorium to be a major site of both conventional and nonconventional secretion of proteins with diverse functions during infection.

## Abstract (precis)

There are many different classes of proteins secreted from Phytophthora infestans that may influence or facilitate infection. Elucidating where and how they are secreted during infection is an important step toward developing methods to control their delivery processes. We used an inhibitor of conventional secretion to identify the following different classes of infection-associated extracellular proteins: cell wall-degrading and cell wall-modifying enzymes, microbe-associated molecular pattern-like proteins that may elicit immune responses, and apoplastic effectors that are predicted to suppress immunity. In contrast, secretion of a cytoplasmic effector that is translocated into host cells is nonconventional, as it is insensitive to inhibitor treatment. This evidence further supports the finding that proteins that are active in the apoplast and effector proteins that are active in the host cytoplasm are differentially secreted by P. infestans. Critically, it demonstrates that a disease-specific developmental structure, the haustorium, is a major secretion site for diverse protein classes during infection.

## INTRODUCTION

Biotrophic pathogens rely on living host cells for growth, deploying a diverse set of secreted virulence determinants to facilitate successful infection (1–4). Over the last three decades, studies of pathogenic bacteria have revealed an arsenal of virulence factors (VFs) involved in pathogenesis, including secreted proteins classed as effectors, protein toxins, and enzymes and cell-surface structures such as capsular polysaccharides, lipopolysaccharides, and outer membrane proteins (5). Filamentous eukaryotic microbial plant pathogens such as fungi and oomycetes secrete a remarkable diversity of molecules at different stages of infection to manipulate the plant immune response (6). In the past 15 years, much research has focused on the activities of cytoplasmic effectors (acting inside host plant cells) and apoplastic effectors (acting in the extracellular space), particularly on where they localize and how they function to facilitate infection. Despite the intense research in this area, many other VFs remain to be characterized.

Phytophthora infestans, causing potato late blight, continues to pose a major threat to potato production worldwide, over 170 years after the Irish potato famine (7). As P. infestans is a model oomycete pathogen, many of its secreted proteins, including cytoplasmic and apoplastic effectors (previously summarized [8, 9]), have been characterized. However, there is still limited knowledge of the majority of secreted proteins representing the repertoire of the P. infestans infection secretome (10). Secreted carbohydrate-active enzymes (CAZymes), including cell wall-degrading enzymes (CWDEs), trypsin-like serine proteases, berberine bridge enzymes, carbonic anhydrases, small cysteine-rich proteins, and repeat-containing proteins, have been postulated to represent potential VFs (10).

Rust fungi and oomycetes produce intercellular hyphae with intracellular haustoria. These structures are formed by enzymatic digestion of the host cell wall and invagination of the host cell membrane. Haustoria create an intimate association with the host cell for molecular exchange, such as nutrient uptake in fungi (11–13). In addition, haustoria are sites for secretion of fungal and oomycete effectors. P. infestans haustorium formation was first examined by electron microscopy (EM) in potato leaf tissue as early as 1966 (14). It was not until 2007 that the first direct observation of effector secretion at haustoria was made (15). Since then, a small number of other cytoplasmic (16, 17) and apoplastic (9) effectors and the transmembrane protein HMP1 (18) have been found to accumulate at *Phytophthora* haustoria.

Conventional and nonconventional forms of secretion of different effector classes were first identified in the rice blast fungus Magnaporthe oryzae. The apoplastic effector “biotrophy-associated secreted protein 4” (Bas4) was conventionally secreted via the endoplasmic reticulum (ER)-to-Golgi secretion pathway from invasive hyphae (IH), as its secretion was sensitive to brefeldin A (BFA; an inhibitor of conventional ER-to-Golgi secretion). In contrast, the cytoplasmic effector Pwl2 (for pathogenicity toward weeping lovegrass) was nonconventionally secreted in a BFA-insensitive manner requiring the exocyst complex (19). In agreement with the study in M. oryzae, two distinct secretory pathways were also found in P. infestans, where the apoplastic effector EPIC1, a cysteine protease inhibitor, was conventionally secreted and the cytoplasmic effector Pi04314 was secreted in a BFA-insensitive manner to accumulate in the host nucleus (9).

We hypothesized that this might be a general rule for oomycete plant pathogens and sought to distinguish conventionally secreted proteins from nonconventionally secreted proteins using liquid chromatography-electrospray tandem mass spectrometry (LC-MS/MS). Although proteomic analysis of extracellular proteins was carried out for P. infestans previously (20), that study did not seek to discriminate between conventional and nonconventional secreted proteins. Here, we performed LC-MS/MS following growth of P. infestans *in vitro* in the presence or absence of BFA to specifically identify inhibitor-sensitive, conventionally secreted candidate VFs. We used a P. infestans transgenic line expressing EPIC1-mRFP (EPIC-monomeric red fluorescent protein), a BFA-sensitive secreted protein (9), to provide a marker for inhibitor activity. We also selected a further cytoplasmic RXLR effector, PITG_22926 (Pi22926), on the basis of its expression profile (21, 22) and its accumulation in the host nucleus to investigate whether translocation from P. infestans into host cells could be observed and whether it was sensitive or insensitive to BFA treatment. Using fusions of mRFP to a range of conventionally and unconventionally secreted proteins, we demonstrate that haustoria are general sites for protein secretion during infection.

## RESULTS

### Identification of a set of proteins that are conventionally secreted *in vitro.*

EPIC1 is a well-characterized apoplastic effector which has been shown to interact with and inhibit secreted plant defense proteases in the apoplast (23). It is secreted via the conventional ER-Golgi secretion pathway in P. infestans (9). To distinguish conventionally secreted proteins from those secreted nonconventionally, culture filtrate (CF) from an EPIC1 transformant constitutively expressing a C-terminally tagged mRFP fusion (EPIC1-mRFP) with a native signal peptide (SP) (9) was used for LC-MS/MS analysis with and without BFA treatment. Fusion protein expression and BFA sensitivity were confirmed by immunoblotting (24), showing that secretion of the EPIC1-mRFP fusion protein was attenuated by BFA treatment compared to the untreated control when grown *in vitro* (see Fig. S1 in the supplemental material).

10.1128/mBio.01216-18.1 FIG S1  Analysis of the efficacy of BFA treatments of the samples of the transformant expressing EPIC1-mRFP (9) used for the LC-MS/MS assay. Download FIG S1, PDF file, 0.2 MB. Copyright © 2018 Wang (王姝梅) et al. 2018 Wang (王姝梅) et al. This content is distributed under the terms of the Creative Commons Attribution 4.0 International license.

LC/MS/MS of the CF ± BFA treatment samples in Fig. S1 identified a total of 421 proteins, peptides of which were detected in at least one biological replicate (see Table S1A in the supplemental material). Of these, 92 genes encoding proteins with predicted signal peptides (SP) were identified using a combination of Phobius version 1.01 (25) and SignalP4.1 (Table S1B). We focused on proteins that (i) contained a secretion SP; (ii) showed enrichment in CF in the absence of BFA (based on log_2_ intensity fold change values corresponding to absence of BFA/presence of BFA ratios of >1); and (iii) were detectable in two or more biological replicates in the absence of BFA. The total number of SP-containing proteins enriched in CF in the absence of BFA was 30 (Table S1C). Nineteen of these SP-containing proteins seen to be enriched in the absence of BFA are predicted to have roles in infection (Table 1). These comprised 6 apoplastic effectors, 6 carbohydrate-active enzymes (CAZymes), 6 candidate microbe-associated molecular patterns (MAMPs), and 1 *in planta*-induced protein (ipiB3) of unknown function (26) (Table 1).

10.1128/mBio.01216-18.7 TABLE S1  (A) List of proteins identified from culture filtrate of Phytophthora infestans transformant expressing EPIC1-mRFP with or without brefeldin A treatment. (B) List of proteins with signal peptide or transmembrane domain. (C) List of proteins enriched in the absence of brefeldin A treatment fractions. Download TABLE S1, XLSX file, 0.3 MB. Copyright © 2018 Wang (王姝梅) et al. 2018 Wang (王姝梅) et al. This content is distributed under the terms of the Creative Commons Attribution 4.0 International license.

**TABLE 1:** Infection-associated secreted proteins enriched in culture filtrate in the absence of BFA^a^

Protein class	Protein ID	Protein name
Apoplastic effectors	D0NBV1	Cystatin-like cysteine protease inhibitor EPIC1
	D0MSS2	Cystatin-like cysteine protease inhibitor EPIC4
	D0MVC9	Kazal-like extracellular serine protease inhibitor EPI1
	D0MVC6	Kazal-like extracellular serine protease inhibitor EPI2
	D0N2T6	Kazal-like extracellular serine protease inhibitorEPI6
	D0NCU6	Small cysteine-rich protein SCR108
		
MAMP-like proteins	D0P3R6	Elicitin-like protein INF4
	D0NUD3	Elicitin-like protein
	D0NKU2	Elicitin-like protein
	D0N3P2	Secretory protein Opel
	D0P1U2	NPP1-like protein
	D0N0V1	NPP1-like protein
		
Carbohydrate-active enzymes (CAZymes)	D0MS99	Pectinesterase PE1
	D0MQ88	Glucan 1,3-beta-glucosidase
	D0NCV1	Glucan 1,3-beta-glucosidase
	D0NSK6	Glycoside hydrolase
	D0N0L9	Glycosyl transferase
	D0NNQ8	1,3-Beta-glucanosyltransferase
		
*In planta*-induced protein	D0NIK4	IpiB3-like protein

a The presence of a signal peptide was detected for all proteins listed in the table. ID, identifier.

Among the apoplastic effectors were EPIC1 and EPIC4, which act in the apoplast to inhibit cysteine proteases (23, 27). Also detected were Kazal-like extracellular serine protease inhibitors EPI2, EPI6, and EPI1, which may play an important role in P. infestans colonization of the host apoplast. EPI1 has been shown to inhibit the tomato pathogenesis-related protease P69B (28). In addition, the small cysteine-rich protein SCR108, like SCR74 and SCR96 (29, 30), potentially influences host interactions.

Candidate MAMPs included 2 elicitin-like proteins that may be recognized by the pattern recognition receptor ELR (elicitin response) from the wild potato Solanum microdontum, which has been shown to detect INF1 (31). In addition, elicitin-like protein INF4, which evades detection by ELR (31), was also enriched in the CF in the absence of BFA. NPP1 (necrosis-inducing *Phytophthora* protein)-like proteins (NLPs) have been found in many plant pathogens, and two were enriched in CF in the absence of BFA treatment. Several members of this protein family can trigger cell death in plants (32, 33). NLPs contain a conserved peptide sequence that acts as a MAMP (34, 35) and is detected by the receptor-like protein RLP23 (36). In addition to elicitin-like proteins and NLPs, a P. infestans homologue of the Opel protein from P. parasitica, which elicits immune responses in *Nicotiana* spp. (37), was also enriched in the absence of BFA.

CAZymes enriched in the absence of BFA included glucan 1,3-beta-glucosidase, pectinesterase (PE), glycosyl transferase, and 1,3-beta-glucanosyltransferase, all of which have been predicted to be phytopathogen virulence factors (10, 38, 39). PE was first purified and characterized from P. infestans CF in 1985, and it was speculated that it contributes to pathogenicity during infection (39). Findings obtained here suggest that there are many conventionally secreted proteins potentially involved in infection that can be detected in CF from *in vitro*-grown P. infestans. We selected a cell wall-degrading enzyme (CWDE), PITG_01029, encoding a pectinesterase (which we renamed PE1), and elicitin-like INF4 for further study to verify their conventional secretion. INF4 was selected as it was reported to evade recognition by the elicitin receptor ELR (31) and may thus potentially be overexpressed in transgenic P. infestans without eliciting immune responses, allowing its secretion during infection to be investigated.

### *INF4* and *PE1* are upregulated during infection and enhance colonization.

To determine whether *INF4* and *PE1* transcripts accumulated to the highest levels during infection, quantitative reverse transcriptase PCR (qRT-PCR) was performed on cDNA derived from mycelium (M), sporangia, and *Nicotiana benthamiana* infected with zoospores of P. infestans isolate 3928A. Infected samples from 12, 24, 33, and 48 h postinfection (hpi) were collected for RNA isolation and cDNA synthesis. The constitutively expressed *actA* gene from P. infestans was used as an endogenous control. *Avr3a*, an RXLR effector from P. infestans which is known to be upregulated during infection (15, 40), was used as a marker of successful infection. Results indicated that the transcript levels of *INF4* and *PE1* at 12 hpi were approximately 1,000- and 100-fold elevated, respectively, compared to expression in the M (normalized to a value of 1 [log value of 0]). *INF4* showed increased transcript abundance relative to the M throughout infection, similarly to *Avr3a* (Fig. S2) (15, 40), whereas the level of expression of *PE1* decreased after 12 hpi (Fig. S2), suggesting that it may particularly contribute to pathogenicity at an early stage of infection. Two further independent biological replicates yielded similar expression profiles (Fig. S2).

10.1128/mBio.01216-18.2 FIG S2  INF4 and pectinesterase (PE) are highly upregulated during infection. Download FIG S2, PDF file, 0.3 MB. Copyright © 2018 Wang (王姝梅) et al. 2018 Wang (王姝梅) et al. This content is distributed under the terms of the Creative Commons Attribution 4.0 International license.

We investigated whether PE1 and INF4 could enhance P. infestans colonization under conditions of transient expression *in planta*, as has been shown for several RXLR effectors previously (see, for example, reference 41). We used Agrobacterium tumefaciens-mediated expression in N. benthamiana of PE1 and INF4 C-terminal fusions with mRFP, including the native signal peptides, followed by inoculation with P. infestans strain 88069. Significantly larger disease lesions were observed in areas expressing PE1-mRFP and INF4-mRFP than in those expressing secreted mRFP (control) at 6 days postinoculation (dpi) (Fig. S3A and S3B). This indicated that both PE1 and INF4 confer a benefit to P. infestans during infection. The stability of expressed fusion proteins was confirmed by immunoblotting (Fig. S3C). As anticipated, in contrast to INF1, INF4 did not elicit cell death when expressed in N. benthamiana (Fig. S3D).

10.1128/mBio.01216-18.3 FIG S3  INF4 and PE enhance P. infestans colonization. Download FIG S3, PDF file, 0.2 MB. Copyright © 2018 Wang (王姝梅) et al. 2018 Wang (王姝梅) et al. This content is distributed under the terms of the Creative Commons Attribution 4.0 International license.

### INF4 and PE1 are conventionally secreted from haustoria.

**FIG 1:**
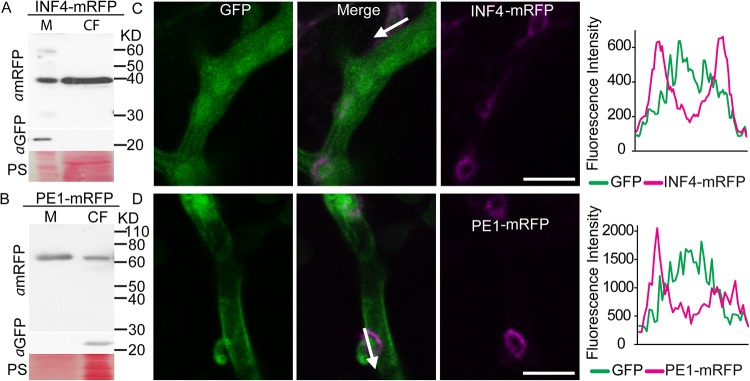
Phytophthora infestans elicitor-like protein INF4 and cell wall-degrading enzyme pectinesterase PE1 are secreted from mycelium into the culture filtrate (CF) *in vitro* and at haustoria *in planta*. (A and B) Ham34 promoter-driven constitutive expression of the INF4 and PE1 fusion proteins tagged with monomeric red fluorescent protein (mRFP) in the mycelium (M) and secretion into the CF of *in vitro*-grown P. infestans were confirmed using an mRFP antibody. Enhanced green fluorescent protein (EGFP; detected with *a*GFP) was used as a marker of cytoplasmic protein, and the results showed that the CF preparation was not detectably contaminated with cellular protein. Size markers are indicated in kilodaltons, and protein loading is indicated by Ponceau staining (PS). (C and D) Confocal projections of P. infestans transformants expressing GFP in the hyphal cytoplasm and INF4-mRFP (C) or PE1-mRFP (D). Secretion of INF4 and PE fusions was observed particularly at haustoria *in planta*. The white arrows show the lines used to generate the fluorescence intensity profiles indicated in the graphs to the right of the images. The *x*-axis data in the graphs represent the distances (in micrometers) from one end of each white arrow in the images to the other end. Scale bars represent 10 µm.

To localize PE1-mRFP and INF4-mRFP during infection, they were transformed into P. infestans. The native signal peptides for secretion were retained. Free green fluorescent protein (GFP) was expressed from the same vector to provide an intracellular control protein and to label hyphae (Fig. S4A). Two independent P. infestans transformants for each construct were used for *in vitro* analysis, showing that PE1-mRFP and INF4-mRFP were largely secreted from the mycelium into the CF. The absence of GFP in the CF indicated there was no detectable intracellular protein contamination of the CF (Fig. 1A and B). Confocal microscopy of N. benthamiana leaf tissue infected with the transformants revealed that secretion of both PE1-mRFP and INF4-mRFP occurred most strongly around the finger-like haustoria (Fig. 1C and D). Fluorescence from the INF4 fusion appeared to envelop the entire haustorium and extended to the surrounding hyphal surface, whereas PE1-mRFP accumulated particularly at the haustorial neck region, where it may help penetration (Fig. 1C and D). This is consistent with results from qRT-PCR, which showed that *PE1* was most highly expressed at an early stage of infection (12 hpi). Thus, the haustorium is a site for secretion not only of the cytoplasmic RXLR effectors and apoplastic effectors (9, 15) but also of the MAMP-like protein INF4 and cell wall-degrading enzyme PE1.

10.1128/mBio.01216-18.4 FIG S4  An independent biological replicate of *in vitro* BFA treatment. Download FIG S4, PDF file, 0.2 MB. Copyright © 2018 Wang (王姝梅) et al. 2018 Wang (王姝梅) et al. This content is distributed under the terms of the Creative Commons Attribution 4.0 International license.

**FIG 2:**
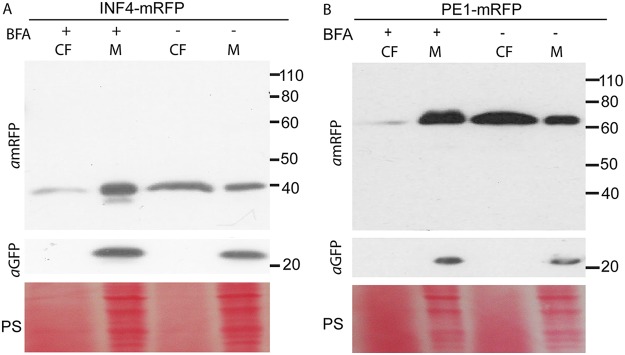
Brefeldin A (BFA) attenuates secretion of a MAMP-like protein and cell wall-degrading enzyme *in vitro*. BFA treatment of Phytophthora infestans transformants constitutively expressing mRFP-tagged INF4 or pectinesterase (PE1) was assayed by immunoblotting. (A) The secretion of INF4-mRFP was attenuated. (B) Secretion of the tagged cell wall-degrading enzyme PE1-mRFP was also sensitive to BFA treatment. αmRFP and *α*GFP were used as primary antibodies to detect mRFP and GFP, respectively. GFP expressed in the cytoplasm of the transformants was used as a cellular protein control to show that there was no detectable contamination of the filtered culture medium (CF) by mycelial (M) proteins. BFA-treated samples are indicated by a plus sign (+), and control samples are indicated by a minus sign (−). Protein size markers are indicated in kilodaltons, and protein loading is shown by Ponceau staining (PS).

These P. infestans transformants were used to investigate secretion during *in vitro* growth, using the inhibitor BFA to confirm that PE1-mRFP and INF4-mRFP were conventionally secreted. In the absence of BFA, the intact fusion proteins were secreted into the CF in each case (Fig. 2). The cellular protein GFP was detected only in the mycelium, indicating negligible contamination of the CF with intracellular proteins. In BFA-treated transformants, secretion of PE1-mRFP and INF4-mRFP was effectively attenuated, with fusion proteins detected primarily in the CF samples not treated with BFA rather than in the CF samples treated with BFA (Fig. 2; see also Fig. S4B and C).

**FIG 3:**
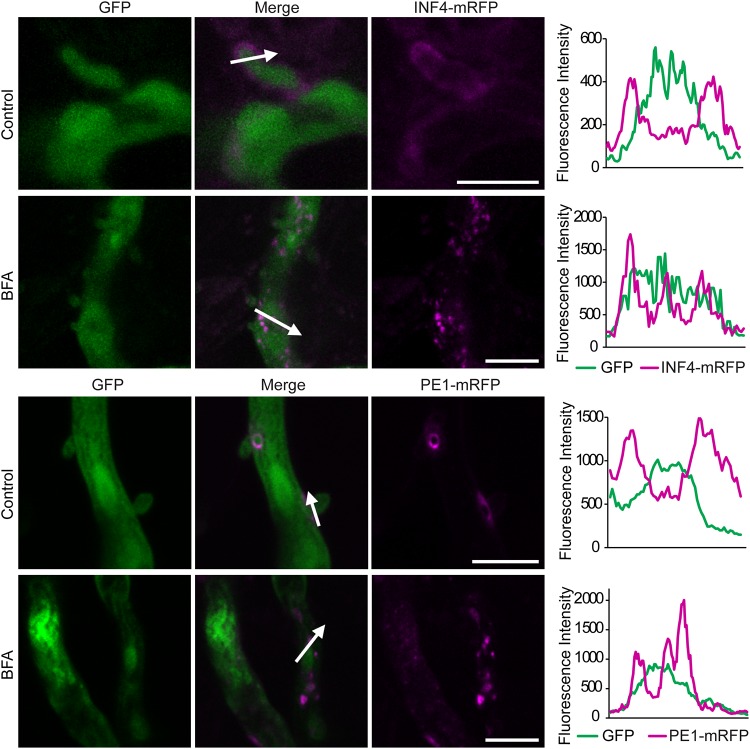
Brefeldin A (BFA) attenuates secretion of INF4 and pectinesterase (PE1) from haustoria *in planta*. Confocal projections of the same transformants used in Fig. 2 in infected *Nicotiana benthamiana* revealed that INF4-mRFP and PE1-mRFP were secreted at haustoria. After BFA treatment, INF4-mRFP and PE1-mRFP were retained in the hyphae and haustoria, accumulating in vesicle-like structures, rather than outlining haustoria as in the water-treated control. White arrows represent the lines used for the fluorescence intensity profiles shown in the graphs to the right of each set of images. The *x*-axis data in the graphs represent the distances (in micrometers) from one end of each white arrow in the images to the other end. Scale bars = 10 µm. The BFA panel was imaged after 24 h of exposure to BFA (50 µg/ml). These images are representative of three independent biological replicates.

In agreement with observations following the *in vitro* BFA sensitivity assay, confocal imaging of infected leaf tissue showed that mRFP fluorescence from P. infestans expressing PE1-mRFP or INF4-mRFP accumulated inside hyphae and haustoria after 24 h of exposure to BFA, suggesting that secretion had been attenuated *in planta* (Fig. 3). Moreover, in the presence of BFA, both PE1-mRFP and INF4-mRFP accumulated in vesicle-like structures (Fig. 3; seen also  in the supplemental material). In total, 11 INF4-mRFP images each were obtained with and without BFA and 8 images each for PE1-mRFP. All images with BFA demonstrated fluorescence accumulation in vesicle-like structures.

### RXLR effector Pi22926 is nonconventionally secreted and translocated from Phytophthora infestans into host plant cells.

To further investigate whether cytoplasmic effectors from P. infestans are nonconventionally secreted from haustoria and delivered into host cells, and as a contrast to the conventionally secreted proteins examined in this work, an additional RXLR effector, Pi22926 (PITG_22926; Fig. S5A), was selected based on its induction during the biotrophic stage of infection (21). RXLR effector Pi22926, expressed transiently in N. benthamiana without SP-encoding sequences as a fusion protein (Pi22926-mRFP), accumulated in the nucleus and nucleolus (Fig. S5B), making it a suitable candidate effector for detecting translocation, as it is clearly detectable in a defined intracellular site in the host cell. The stability of the fusion protein was confirmed by immunoblotting (Fig. S5C).

10.1128/mBio.01216-18.5 FIG S5  Phytophthora infestans RXLR effector Pi22926 localizes to the nucleus and accumulates in the nucleolus. Download FIG S5, PDF file, 0.2 MB. Copyright © 2018 Wang (王姝梅) et al. 2018 Wang (王姝梅) et al. This content is distributed under the terms of the Creative Commons Attribution 4.0 International license.

**FIG 4:**
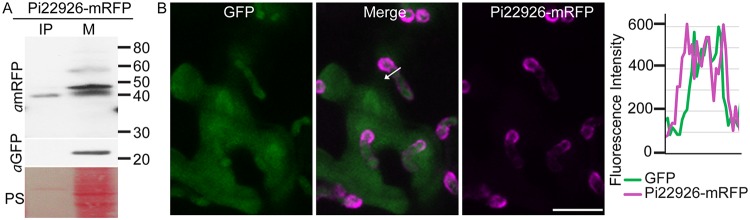
Phytophthora infestans RXLR effector 22926 (Pi22926) is secreted from mycelium *in vitro* and localizes to haustoria during infection. (A) Expression of the Pi22926-monomeric red fluorescent protein (mRFP) fusion protein was confirmed using an mRFP antibody. Fusion protein was detected in *in vitro*-grown mycelium (M) and was IP from infected tissue by P. infestans transformants constitutively expressing Pi22926-mRFP fusions. Green fluorescent protein antibody (*α*GFP) was used as a cytoplasmic marker as described for Fig. 2. Size markers are indicated in kilodaltons, and protein loading is indicated by Ponceau staining (PS). (B) Confocal projection of intercellular hyphae and haustoria of the Pi22926-mRFP transformant in infected N. benthamiana leaf tissue, showing that the fusion protein was present at haustoria. The white arrow indicates the path used for the fluorescence intensity profile of mRFP and GFP fluorophores across the haustorium. The profile is shown at the right of the images. The *x*-axis data in the graphs represent the distance (in micrometers) from one end of the white arrow to the other end. Scale bar represents 10 µm.

To observe whether Pi22926 was secreted at haustoria and delivered into host nuclei, we generated P. infestans transformants expressing SP-Pi22926-mRFP and coexpressing free GFP in the pathogen cytoplasm for labeling hyphae. Expression of SP-Pi22926-mRFP and GFP was driven by independent copies of the constitutive Ham34 promoter (Fig. S6A). Protein extraction and immunoblotting from *in vitro*-grown transgenic P. infestans showed that there were two bands from effector-mRFP fusions in the mycelium sample, the smaller of which was evident when immunoprecipitated (IP) from infected leaf material (Fig. 4A). No Pi22926-mRFP was detected in CF samples from *in vitro* growth, suggesting that the protein fusion was not stable under these conditions. GFP was detected in the mycelium fraction but was not detected in the IP from infected material, indicating that cytoplasmic proteins did not contaminate the mRFP_Trap beads.

10.1128/mBio.01216-18.6 FIG S6  Confocal projection of an additional transformant expressing SP-Pi22926-mRFP. Download FIG S6, PDF file, 0.3 MB. Copyright © 2018 Wang (王姝梅) et al. 2018 Wang (王姝梅) et al. This content is distributed under the terms of the Creative Commons Attribution 4.0 International license.

**FIG 5:**
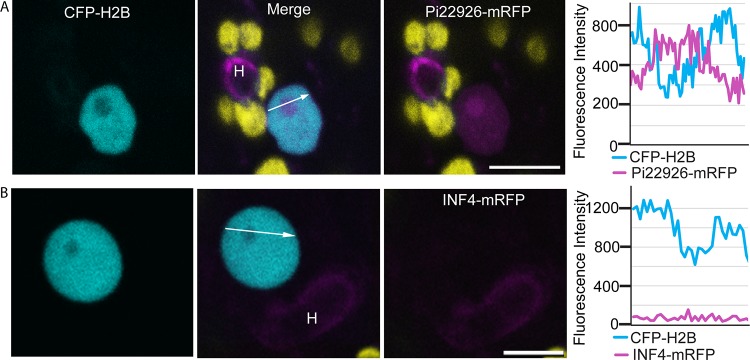
Phytophthora infestans effector Pi22926 tagged with monomeric red fluorescent protein (Pi22926-mRFP) was translocated from the pathogen haustoria to the host nucleus and nucleolus. (A) Representative confocal projection of a haustoriated (H) N. benthamiana cell, in which the nucleus was labeled by CFP-NbH2B. Red fluorescence was observed in the nucleolus and nucleoplasm, indicating that Pi22926 fusion proteins have translocated from haustoria into the host cells. (B) Confocal projection of a typical nucleus from a leaf infected by the transformant expressing INF4-mRFP. No red fluorescence was detected in the nucleus. Chloroplast autofluorescence is indicated in yellow. The white arrows show the lines used for the fluorescence intensity profiles indicated in the graphs to the right of the image sets. The *x*-axis data in the graphs represent the distances (in micrometers) from one end of each white arrow in the images to the other end. These images are representative of 46 images of haustoriated cells from different independent biological replicates with four independent transformants. Bars, 10 µm.

During infection, fluorescence from the Pi22926-mRFP fusion protein outlined the haustorium (Fig. 4B), further confirming that the haustorium is a major secretion site during infection. Importantly, confocal microscopy revealed that Pi22926-mRFP fusion protein from two independent P. infestans transformants was detectable in nuclei of haustoriated host cells, where it accumulated in the nucleolus in each case (Fig. 5A; see also Fig. S6B). From three independent biological replications for each transformant, a total of 42 haustoriated plant cells were examined, and mRFP fluorescence was observed in the nuclei in 40 of the 42 haustoriated host cells. This is in agreement with our previous observations obtained with RXLR effector Pi04314 (9). In contrast, no mRFP fluorescence was detected inside haustoriated host cells infected by transgenic lines expressing SP-INF4-mRFP (an example is shown in Fig. 5B).

**FIG 6:**
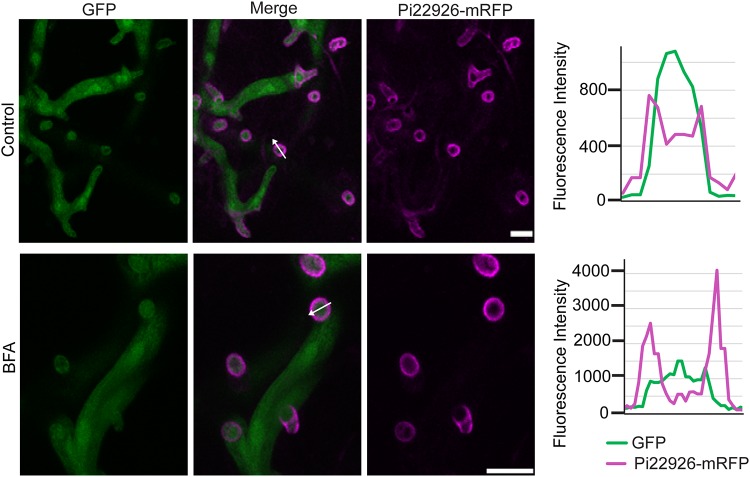
Secretion of the Phytophthora infestans cytoplasmic effector Pi22926 is insensitive to brefeldin A (BFA) during infection. Confocal projections of the Pi22926-mRFP-expressing transformant in infected N. benthamiana reveal that the fusion protein is preferentially secreted at haustoria even in the presence of BFA. White arrows indicate the lines used for the fluorescence intensity profiles shown in graphs to the right of each image. The *x*-axis data in the graphs represent the distances (in micrometers) from one end of each white arrow in the images to the other end. Scale bars represent 10 µm. The BFA panel was imaged after 24 h of exposure to BFA. These images are representative of more than 100 haustoria each either with or without BFA treatment from three independent biological replicates.

To investigate whether secretion of Pi22926 was nonconventional, we treated infected leaf tissue with BFA. Confocal microscopy revealed that Pi22926-mRFP fluorescence still accumulated around haustoria following 24 h of exposure of infected N. benthamiana leaves to BFA (Fig. 6). This is consistent with the BFA-insensitive secretion of Pi04314 as reported previously (9) and further supports observations that cytoplasmic effectors and apoplast-active proteins are secreted from P. infestans haustoria via different pathways.

## DISCUSSION

In this study, we identified candidate conventionally secreted proteins by LC-MS/MS analysis of the secreted proteome in the presence or absence of treatment with the ER-to-Golgi inhibitor BFA. This strategy revealed 19 P. infestans proteins with secretion signal peptides (SPs) that were enriched in extracellular medium in the absence of BFA and that are predicted to influence host plant interactions. These included apoplastic effectors, which suppress host defenses; elicitin-like proteins, NLPs, and Opel, which may elicit immune responses in the host; and CAZymes, which might change the biochemical characteristics of the host and pathogen cell walls to promote disease progression. We previously characterized the secretion of the typical apoplastic effector EPIC1, showing that it was sensitive to BFA (9). Therefore, we widened our study to a MAMP-like protein, INF4, and a cell wall-degrading enzyme, pectinesterase PE1, in this work. We showed that secretion of INF4 and PE1 is also sensitive to BFA and occurs at haustoria. This suggests the general rule that apoplast-active proteins are conventionally subject to ER-to-Golgi secretion. Moreover, and again in agreement with our earlier report (9), we show that a second cytoplasmic RXLR effector, Pi22926, is nonconventionally secreted from haustoria to be delivered to the host nucleus. These results are discussed below.

We observed that secreted PE1 localized specifically around the base of haustoria. As PE1 has a pectin-binding domain, this potentially reflects binding to the plant cell wall. PE proteins are major enzymes that facilitate plant cell wall modification and subsequent breakdown. Many pathogens use PE proteins to macerate the cell wall and gain access to plant cells (42). Overexpression of PE1 enhanced P. infestans colonization of N. benthamiana, suggesting that PEs play a critical role early in infection, softening cell walls to enable P. infestans to develop haustoria, leading to intimate contact with the host cell membrane.

Oomycetes are sterol auxotrophs, and elicitins have been shown to act as sterol carrier proteins (43), presumably to facilitate capture of sterols from the environment. Interestingly, some elicitins, such as INF1, are downregulated during infection (44, 45). This likely reflects the fact that they are targeted as MAMPs for recognition by host receptors (31). However, INF4 evades recognition by the elicitin receptor-like protein ELR (31). Here we confirmed that INF4 was not recognized in N. benthamiana (see Fig. S4D in the supplemental material) and so is unlikely to act as a MAMP. INF4 may have coevolved to escape host detection by receptors such as ELR but to retain its function in scavenging sterols, allowing this essential function to proceed during infection. These possibilities were not investigated further here but could be tested through a strategy such as mutagenesis of specific residues in INF4 to determine their impact on pathogenicity or sterol binding.

The haustorium is emerging as a major site of secretion of cytoplasmic RXLR effectors and apoplastic effectors and is the location of the transmembrane protein Hmp1, which is essential for infection (9, 15–18). To demonstrate the importance of the haustorial interface as a battleground between pathogen and host, a wide range of secreted proteins need to be studied. Here, we showed that PE1 was secreted from haustoria (Fig. 1D), consistent with previous studies providing evidence for host cell wall degradation by P. infestans at sites of haustorium formation (46, 47). Interestingly, whereas INF4 was localized in the extrahaustorial matrix, PE1 was localized specifically as a ring toward the base of the haustorium. The localization of the MAMP-like INF4 to haustoria (Fig. 1C) suggests that this is a likely site of (MAMP)-triggered immunity (PTI) activation (48) and that it is, therefore, vital to suppress PTI responses using effectors also secreted at this location (9, 15). This is the first observation of the secretion and localization of a MAMP-like protein and a cell wall-degrading enzyme involved in P. infestans pathogenesis.

RXLR effector Pi22926 was secreted from haustoria (Fig. 4) and accumulated in plant cell nuclei (Fig. 5), indicating that it was translocated into host cells as anticipated. Two protein bands were detected in mycelium samples, the smaller of which was evident during infection. As indicated for Avr3a by Wawra et al. (49), the different sizes of fusion protein could be explained by proteolytic cleavage of the RXLR domain. The predicted size of Pi22926-mRFP after SP removal is 47.8 kDa, whereas cleavage at the RXLR domain would reduce the size of the fusion protein by 2.92 kDa to 44.88 kDa. Further investigation with transformants in which the RXLR motif has been mutated will be needed to investigate whether proteolytic cleavage occurs.

### Conclusion.

PE1 and INF4 were enriched in the absence of BFA treatment in the P. infestans secretome. Indeed, secretion of both PE1-mRFP and INF4-mRFP in P. infestans transformants was attenuated by BFA treatment (Fig. 2 and 3). This confirms that they are conventionally secreted VFs. The observation of haustorial secretion of a MAMP-like protein and a cell wall-degrading enzyme together with cytoplasmic effector Pi22926 and previously identified secreted proteins (9, 15, 16, 18) provides strong evidence that the haustorium is a major site of general secretion during infection. This report brings new insight into virulence determinants in P. infestans infection and suggests that secretion processes may provide good targets for chemical control of this economically devastating crop disease.

## MATERIALS AND METHODS

### *Phytophthora* infestans culture and *Nicotiana benthamiana* inoculation.

Phytophthora infestans wild-type strains 88069 and 3928 A and transgenic lines were cultured as described by Grenville-Briggs et al. (50). Infection inoculum was prepared and placed in 10-µl droplets onto plant leaves as described by Whisson et al. (15). Samples for qRT-PCR were collected as 4.5-mm-diameter discs from inoculation sites at 12, 24, 36, and 48 hpi. Nonadhering and ungerminated spores were removed by rinsing the leaf discs with sterile water.

### Phytophthora infestans transformation vector construction and transformation.

The Ham34 promoter (Ham34P) was cloned from oomycete expression vector pTOR (GenBank accession number EU257520). Gene-specific primers (see Table S2 in the supplemental material) modified to contain restriction enzyme recognition sites were used in PCRs. PCR products were purified and digested with KpnI restriction endonuclease and ligated into the same restriction site in plasmid pPLRAG (9) to yield Ham34P-pPLRAG (pHPRAG). P. infestans genes encoding INF4 (PITG_21410), pectinesterase (PITG_01029, PE1), and Pi22926 were cloned, retaining the native predicted signal peptides. Gene-specific primers (Table S2) with restriction enzyme recognition sites were used to amplify the genes from genomic DNA of isolate 3928A. PCR products were purified, digested with NotI restriction endonuclease, and ligated into the same restriction sites in plasmid pHPRAG to generate pHPRAG _INF4, pHPRAG _PE1, and pHPRAG _Pi22926. Transformation of P. infestans was carried out using a modified polyethylene glycol (PEG)-CaCl_2_-lipofectin protocol (51), modified as described by Avrova et al. (18). P. infestans transformants were maintained in the dark at 19°C on RyeA agar supplemented with 20 µg/ml geneticin antibiotic.

10.1128/mBio.01216-18.8 TABLE S2  Oligonucleotide primers for qRT-PCR and constructs for P. infestans transformation. Download TABLE S2, DOCX file, 0.01 MB. Copyright © 2018 Wang (王姝梅) et al. 2018 Wang (王姝梅) et al. This content is distributed under the terms of the Creative Commons Attribution 4.0 International license.

### Vector construction and Agrobacterium tumefaciens transient assays.

P. infestans genes encoding INF4, PE1, and Pi22926 were amplified, retaining the native predicted signal peptide and C-terminal mRFP from pHPRAG_INF4, pHPRAG_PE1, and pHPRAG_Pi22926, respectively. Gene-specific primers that included flanking Gateway recombination sites at both termini were used in nested PCR (Table S2). Purified PCR products were recombined into pDONR201 (Invitrogen) to generate entry clones. The entry clones with genes of interest were recombined with pB2GW7 and electroporated into Agrobacterium tumefaciens strain AGL1 for expression of fusion proteins *in planta* (52). A. tumefaciens transient-transformation assays (ATTAs) were performed as described by Wang et al. (9). Hypersensitive response (HR) analyses were carried out by infiltrating agrobacteria mediating expression of the INF1 construct as positive control or INF4-mRFP at an OD_600_ of 0.5. The degree of HR was assessed at 4 and 8 dpi as previously described (16).

### Quantitative RT-PCR analysis.

RNA was extracted using a Qiagen RNeasy plant minikit (Qiagen, Crawley, United Kingdom), according to the manufacturer’s instructions. DNA contamination was removed using a Turbo DNA-free kit (Ambion) based on the manufacturer’s protocol. First-strand cDNA was synthesized from 5 µg of total RNA by oligo(dT) priming using Superscript II RNaseH reverse transcriptase (Invitrogen Life Technologies, Inc. Ltd., Paisley, United Kingdom) following the manufacturer’s recommendations. Power SYBR green master mix (Applied Biosystems, Foster City, CA) was used for real-time qRT-PCR, which was performed on a Chromo4 thermal cycler (MJ Research, United Kingdom) using Opticon Monitor 3 software with the following program: 95°C for 15 min followed by 40 cycles for quantitative PCR of 95°C for 15 s and 60°C for 1 min and, finally, melting curve analysis from 58°C to 95°C. The primer sequences used in the reactions are listed in Table S2. Data analysis was carried out as described by Avrova et al. (53). In each case, the constitutively expressed P. infestans *actA* gene was used as the internal control.

### Western blotting and immunoprecipitation.

P. infestans transformants were cultured in amended lima bean (ALB) liquid medium containing 20 µg/ml geneticin antibiotic (54). Mycelium was separated after 2 dpi using a 70-µm-pore-size filter. To test the effects of BFA on protein secretion *in vitro*, the method described previously by Wang et al. (9) was followed. Briefly, mycelia of transformants expressing INF4-mRFP, PE1-mRFP, and Pi22926-mRFP were incubated for 24 h in the dark at 19°C in 1 ml of ALB liquid medium containing 20 µg/ml geneticin and 50 µg/ml BFA (5 mg/ml in a dimethyl sulfoxide [DMSO] stock solution of BFA was diluted in water to 50 µg/ml). To reduce background levels of plant proteins in the medium for LC-MS/MS analysis, EPIC1-mRFP was incubated in 1 ml of diluted ALB by the use of modified minimum medium (1:1 [vol/vol]) based on the method of Leal et al. (55) as follows. Briefly, 5 g d-glucose; 0.5 g KH_2_PO_4_; 0.5 g MgSO_4_; 0.5 mg FeSO_4 ⋅ _7H_2_O and ZnSO_4 ⋅ _7H_2_O; 0.02 mg CuSO_4 ⋅ _5H_2_O, MnCl_2 ⋅ _4H_2_O, and Na_2_MoO_4 ⋅ _2H_2_O; 1 mg thiamine hydrochloride; 10 mg β-sitosterol; 4.52 g methionine; and 4.03 g aspartate were combined and double-distilled water (ddH_2_O) was added to reach a final volume of 1 liter. The PH was adjusted to 7 by addition of 1 M KOH, and then the reaction mixture was autoclaved for 20 min at 121°C. Amino acids were sterilized by the use of a 0.22-µm-pore-size filter and were added after autoclaving. The culture filtrate (CF) was retained separately after removing mycelia, and four times the sample volume of cold (−20°C) acetone (Thermo Fisher Scientific, Loughborough, United Kingdom) was used to precipitate proteins overnight. Protein was collected by centrifugation at 10,000 × *g* for 10 min at 4°C. One hundred milligrams of P. infestans mycelium was ground in liquid nitrogen (LN2) and resuspended in the same volume of 2% sodium dodecyl sulfate-polyacrylamide gel electrophoresis (SDS-PAGE) sample-loading buffer (100 mM Tris, 4% SDS, 20% glycerol, 0.2% bromophenol blue). To immunoprecipitate mRFP-tagged Pi22926 from N. benthamiana leaves that were infected by a transformant expressing Pi22926-mRFP, proteins were extracted using GTEN buffer (10% [vol/vol] glycerol, 25 mM Tris-HCl [pH 7.5], 1 mM EDTA, 150 mM NaCl) combined with 10 mM dithiothreitol (DTT), protease inhibitor cocktail, 1 mM phenylmethyl sulfonyl fluoride, and 0.2% Nonidet P-40 and were then incubated with mRFP-Trap_M beads (Chromtek) for 90 min at 4°C. Beads were washed three times in GTEN buffer containing protease inhibitor cocktail and 1 mM phenylmethyl sulfonyl fluoride and were resuspended with 2× SDS-PAGE sample-loading buffer (56). Samples were loaded onto a 12% bis-Tris NuPAGE Novex gel (Invitrogen). Gel electrophoresis and membrane blocking and washing steps were performed as described by McLellan et al. (57). Primary antibodies *a*mRFP and *a*GFP (Sigma-Aldrich) were used at a 1:4,000 dilution. Secondary antibodies anti-rat immunoglobulin G (IgG) horseradish peroxidase (HRP) and anti-mouse IgG HRP (Sigma-Aldrich) were used at 1:5,000 dilutions. ECL substrate (Thermo Scientific Pierce, Rockford, IL) was used to detect protein bands on immunoblots, following the manufacturer’s protocol.

### LC-MS/MS analysis.

Secreted protein mixtures were digested by the use of 1 µg/µl Trypsin Gold (reconstituted with 50 mM acetic acid) at a 1:100 enzyme-to-substrate (microgram) ratio. Digested peptides were run on an LTQ-Orbitrap XL instrument (Thermo Fisher, United Kingdom) coupled to a Dionex UltiMate 3000 high-performance liquid chromatography (HPLC) system (Thermo Fisher Scientific, United Kingdom). Eluent A (3% acetonitrile–0.05% formic acid) and eluent B (80% acetonitrile–0.04% formic acid) were used for a 2% to 40% gradient. The top 7 most intense peaks in each MS1 scan were then taken for MS2 analysis. The level of resolution for MS2 spectra was 6,000, and the spectra were fragmented using collision-induced dissociation (CID) and a mass range of 335 to 1,800 *m*/*z*. MS/MS data were analyzed using Maxquant version 1.6.0.16 against the P. infestans T30-4 database from Uniprot, the MS/MS–flow-injection mass spectrometry (FIMS) tolerance was set to 20 ppm, and the ion trap mass spectrometer (ITMS) match tolerance was 0.15. Fixed modifications included carbamidomethyl (C), and variable modifications included oxidation (M); a maximum value of 2 missed cleavages was used. The protein false-discovery rate (FDR) was set to 0.01. The minimum peptide length was 7. Output data from Maxquant were filtered by the use of Perseus_1.5.6.0, peptide intensity data were transformed and used for *P* value analysis, and a two-way *t* test was used for analyzing differentially expressed proteins in the two samples being compared. Signal peptide (SP) and transmembrane prediction was performed using Phobius version 1.01 (25) and SignalP 4.1 (58) on amino acid sequences. hmmsearch version 3.1b1 (59) (PFAM release 31.0 [24 February 2017]) was used to identify PFAM domains within amino acid sequences. Gathering cutoffs (--cut_ga) was applied to hmmsearch to reduce levels of false positives.

### Confocal microscopy.

N. benthamiana leaf pieces infected with biotrophy-stage P. infestans were mounted on slides and imaged using a Nikon A1R confocal microscope with a 40×/0.8 water dipping lens. Enhanced green fluorescent protein (EGFP) was imaged with 488-nm excitation, and emissions were collected at wavelengths between 500 and 530 nm. Cyan fluorescent protein (CFP) was imaged with 456-nm excitation, and emissions were collected at 480 nm. Imaging of mRFP was conducted using 561-nm excitation, and emissions were collected at wavelengths between 600 and 630 nm. The pinhole was set to 1 Airy unit for the fluorophore with the longest wavelength. Single optical-section images and z-series were collected from leaf tissue that was not heavily colonized by P. infestans to minimize the potential for interference from autofluorescence from the cell damage and death caused by infection. For BFA treatments *in planta*, 50 µg/ml BFA–water was infiltrated into leaf tissue infected by transformants (9). Treated leaves were imaged after 24 h.

### Data availability.

The mass spectrometry proteomics data have been deposited in the ProteomeXchange Consortium via the PRIDE (60) partner repository with the data set identifier PXD009802.

### Accession number(s).

UniProt protein accession numbers are listed in Table 1.

10.1128/mBio.01216-18.9 MOVIE S1  INF4-mRFP fluorescence during infection following treatment with BFA. Download MOVIE S1, AVI file, 14 MB. Copyright © 2018 Wang (王姝梅) et al. 2018 Wang (王姝梅) et al. This content is distributed under the terms of the Creative Commons Attribution 4.0 International license.

10.1128/mBio.01216-18.10 MOVIE S2  PE1-mRFP fluorescence during infection following treatment with BFA. Download MOVIE S2, AVI file, 14 MB. Copyright © 2018 Wang (王姝梅) et al. 2018 Wang (王姝梅) et al. This content is distributed under the terms of the Creative Commons Attribution 4.0 International license.
