# Supplementary material for: The Phytophthora infestans Haustorium Is a Site for Secretion of Diverse Classes of Infection-Associated Proteins
Source: mBio. 2018 Aug 28;9(4):e01216-18. doi: 10.1128/mBio.01216-18 (PMC6113627; doi:10.1128/mBio.01216-18)
Supplement: FIG S1 [file mbo004184040sf1.pdf]

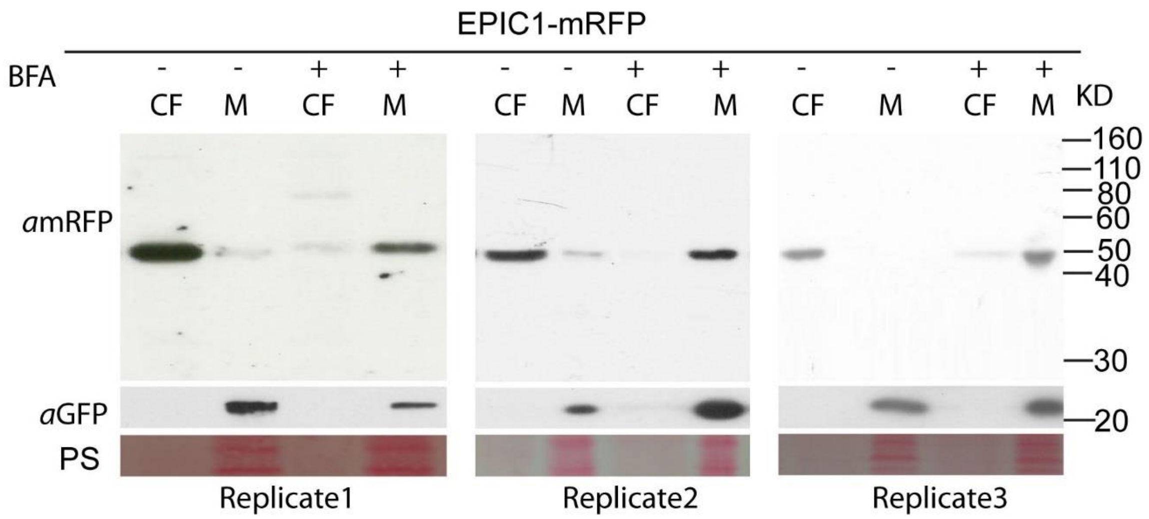

FIG S1 Analysis of the efficacy of BFA treatments on the samples of the transformant expressing EPIC1-mRFP (9) used for the LC-MS/MS assay. Immunoblotting was carried out to show BFA treatment (+) blocks secretion of EPIC1-mRFP by the conventional secretory pathway in three independent biological replicates. Green fluorescent protein was used as marker of cytoplasmic protein and showed that the CF preparation was not detectably contaminated with cellular protein.
