# Supplementary material for: The Phytophthora infestans Haustorium Is a Site for Secretion of Diverse Classes of Infection-Associated Proteins
Source: mBio. 2018 Aug 28;9(4):e01216-18. doi: 10.1128/mBio.01216-18 (PMC6113627; doi:10.1128/mBio.01216-18)
Supplement: FIG S2 [file mbo004184040sf2.pdf]

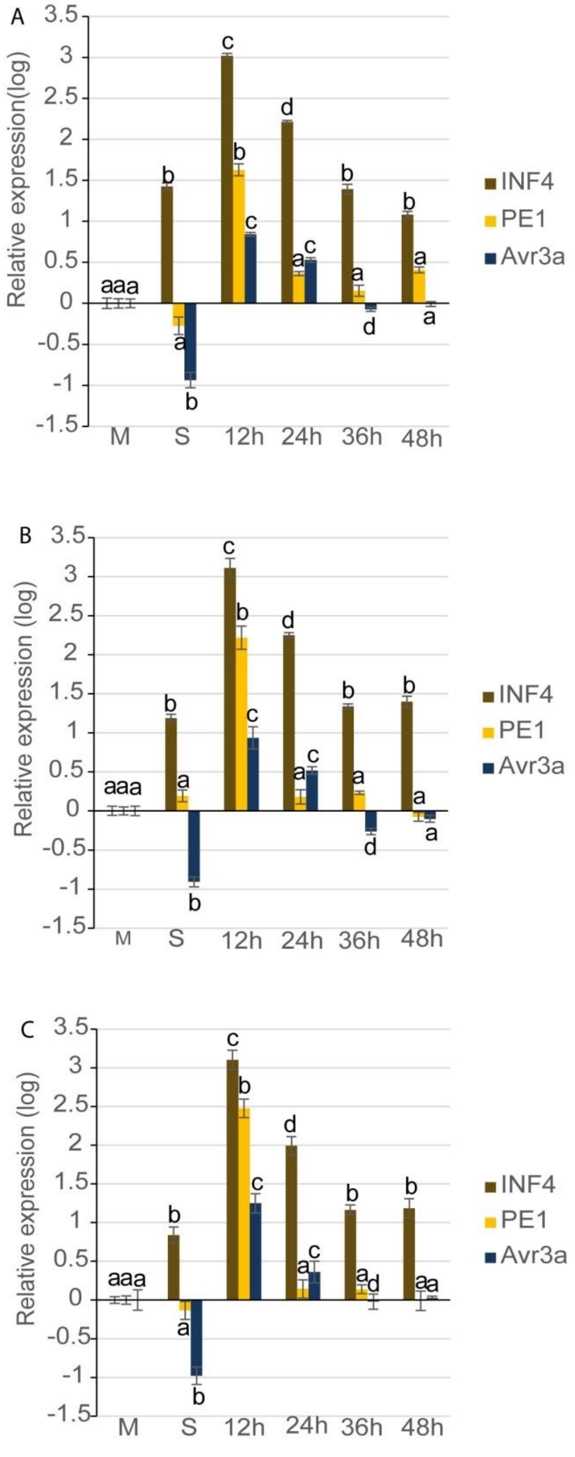

FIG S2 INF4 and Pectinesterase (PE) are highly up-regulated during infection.

Quantitative RT-PCR shows that the relative expression levels of INF4 (PITG_21410) and pectinesterase (PE1, PITG_01029), like the effector Avr3a (PITG_14783), were dramatically up-regulated at 12h post infection (hpi) compared to *in vitro* mycelium (M) levels, which were normalized to log1 (0). *INF4* remained highly up-regulated beyond 12 hpi whereas the level of expression of *PE1* decreased relative to M, but is still high relative to S. S=Sporangia. Error bars are standard error. A, B, C are three independent biological replications. Letters on the graph denote statistically significant differences ($p < 0.05$ ANOVA, Student-Newman-Keuls).
