# Supplementary material for: The Phytophthora infestans Haustorium Is a Site for Secretion of Diverse Classes of Infection-Associated Proteins
Source: mBio. 2018 Aug 28;9(4):e01216-18. doi: 10.1128/mBio.01216-18 (PMC6113627; doi:10.1128/mBio.01216-18)
Supplement: FIG S3 [file mbo004184040sf3.pdf]

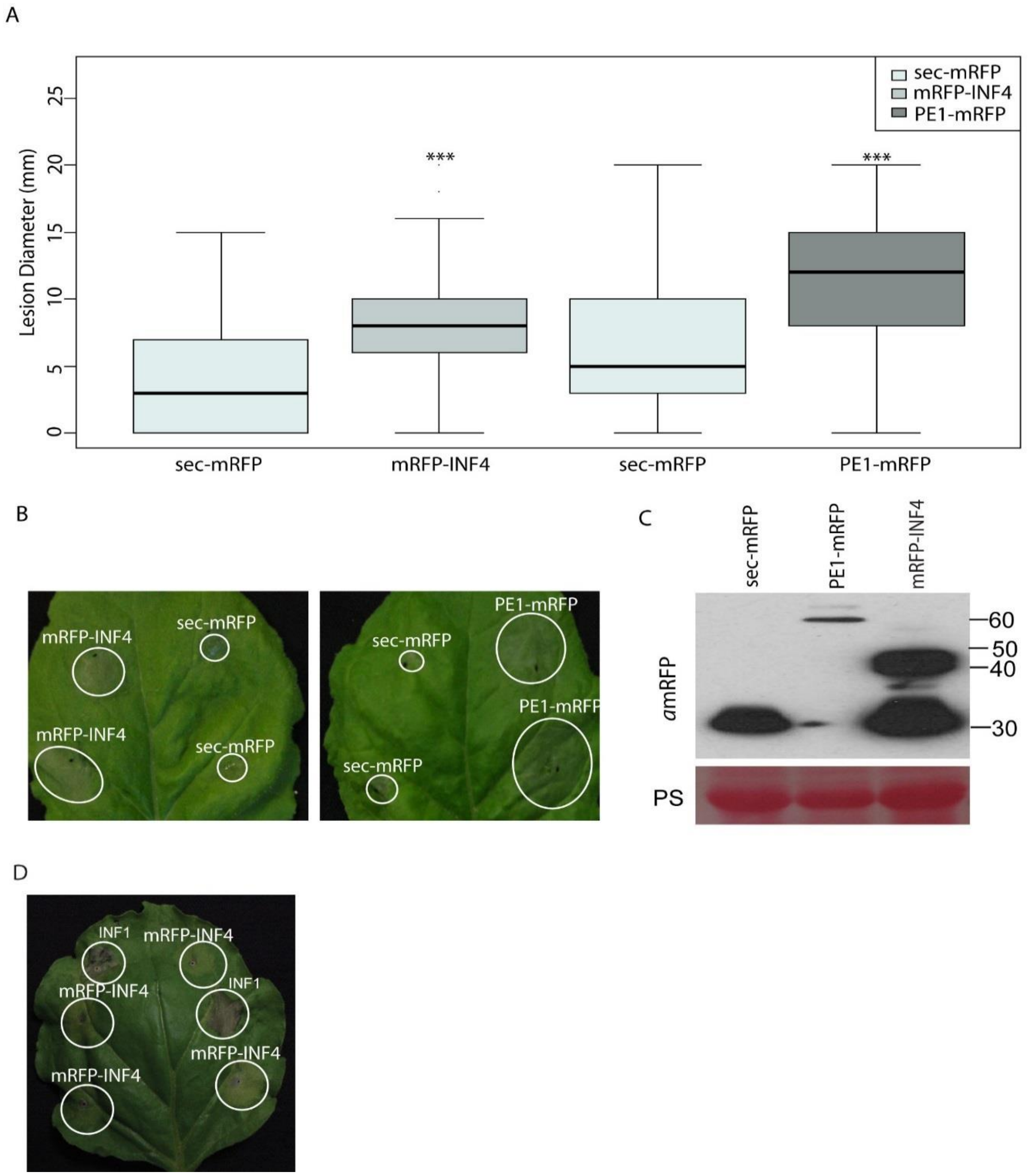

FIG S3 INF4 and PE enhance *P. infestans* colonization.

(A) The boxplot shows a significant increase in the diameters of *P. infestans* disease lesions following infection of areas on *N. benthamiana* leaves transiently expressing INF4-mRFP and PE1-mRFP compared to the secreted mRFP control (ANOVA, $p \leq 0.001$ ***, n = 72). (B) Images of infected leaves shows the extent of *P. infestans* colonization with INF4-mRFP or PE1-mRFP compared to the secreted mRFP control sites. (C) The immunoblotting shows the stability of proteins used in (A), using an mRFP antibody. Size marker is indicated in kDa, and protein loading is indicated by Ponceau stain (PS). (D) Tissue death responses are triggered in *N. benthamiana* by transient expression of INF1 but not by the mRFP-INF4 fusion. Leaves were photographed 5 d after infiltration. White circles highlight infiltrated areas.
