# Supplementary material for: The Phytophthora infestans Haustorium Is a Site for Secretion of Diverse Classes of Infection-Associated Proteins
Source: mBio. 2018 Aug 28;9(4):e01216-18. doi: 10.1128/mBio.01216-18 (PMC6113627; doi:10.1128/mBio.01216-18)
Supplement: FIG S4 [file mbo004184040sf4.pdf]

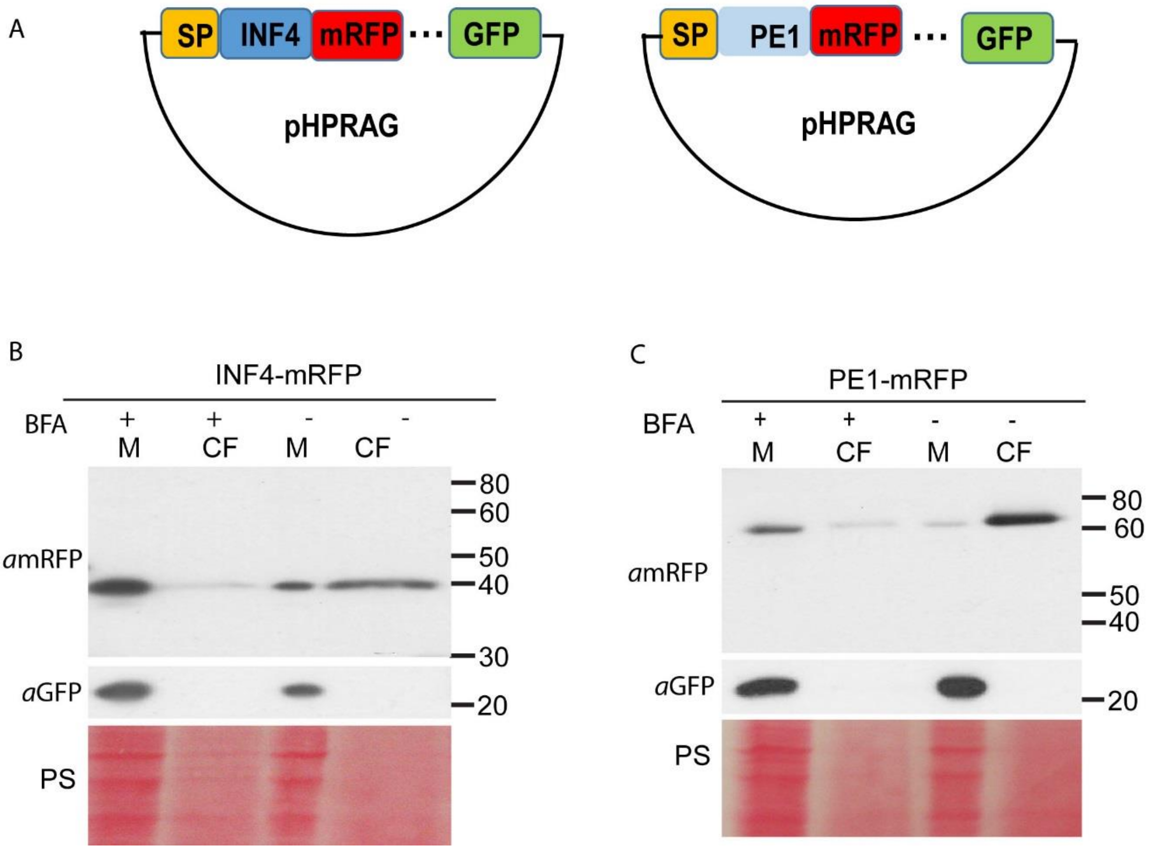

FIG S4 An independent biological replicate of *in vitro* BFA treatment.

(A) A diagram of the constructs for *P. infestans* transformation to express the SP-INF4-mRFP and SP-Pectinesterase (PE1)-mRFP fusion proteins. The native signal peptides (SP) were included. GFP was expressed from a separate promoter on the same plasmid to label the hyphal cytoplasm. (B-C) BFA treatment (+) inhibits secretion of INF4 -mRFP (B) and PE1-mRFP (C) into the culture filtrate (CF), indicating the BFA effect on INF4 -mRFP and PE1-mRFP is consistent.
