# Supplementary material for: The Phytophthora infestans Haustorium Is a Site for Secretion of Diverse Classes of Infection-Associated Proteins
Source: mBio. 2018 Aug 28;9(4):e01216-18. doi: 10.1128/mBio.01216-18 (PMC6113627; doi:10.1128/mBio.01216-18)
Supplement: FIG S5 [file mbo004184040sf5.pdf]

A

MLRSFLLIVATVSLFGQCKPLPLATSPVSDAVRAPHRSTHETRFLRTNDEERGATMTL
AGVLRDKAQTKQLLTSWLNSGKSVPSVSNKLGLKRMSLEQAIHHENWKALTTFQR
MKSKKAKAYAKYGTGYQTEAKTKENLLQWVMRGDSPKEVSSTLGLLGLSRRKIID
HQNYEAFRTFLKYRKQWAEMQGNGFTKLTT

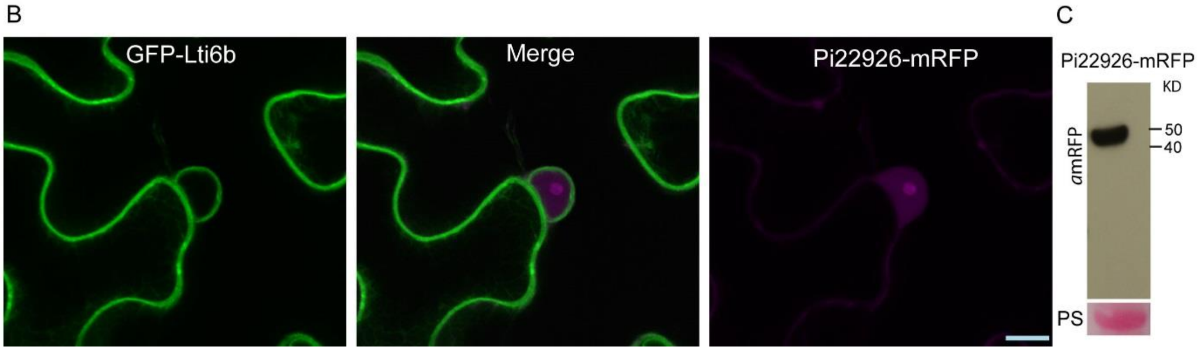

FIG S5 *Phytophthora infestans* RXLR effector Pi22926 localises to the nucleus and accumulates in the nucleolus. (A) The protein sequence of Pi22926. RXLR and EER motifs are indicated in red. The signal peptide is highlighted in yellow. (B) Single optical section confocal image of the Pi22926-mRFP fusion protein expressed in transgenic *N. benthamiana* in which the plasma membrane and nuclear membrane were labelled with GFP-LTi6b. The Pi22926-mRFP fusion localises to the nucleoplasm and nucleolus with faint cytoplasmic signal. Scale bar indicates 10 µm. (C) Immunoblot of the construct used in (B), showing the stability of the fusion protein.
