# Supplementary material for: The Phytophthora infestans Haustorium Is a Site for Secretion of Diverse Classes of Infection-Associated Proteins
Source: mBio. 2018 Aug 28;9(4):e01216-18. doi: 10.1128/mBio.01216-18 (PMC6113627; doi:10.1128/mBio.01216-18)
Supplement: FIG S6 [file mbo004184040sf6.pdf]

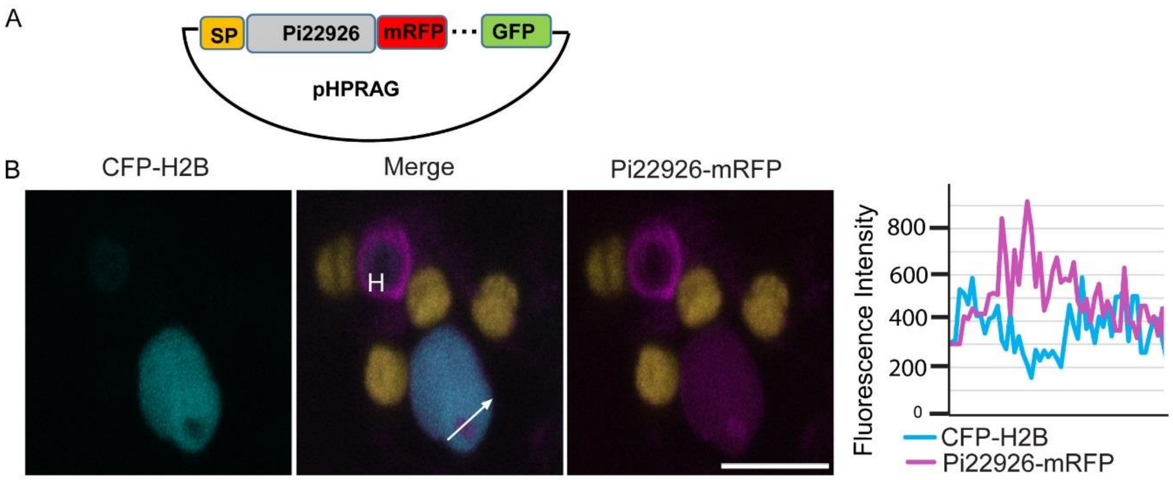

FIG S6 Confocal projection of an additional transformant expressing SP-Pi22926-mRFP.

(A) A diagram of the construct used for *P. infestans* transformation to express the SP-Pi22926-mRFP fusion protein and cytoplasmic GFP. (B) Confocal projection confirming that Pi22926-mRFP translocation from the transformed pathogen into host cells is a consistent behaviour in haustoriated cells. White arrow indicates the line used for the fluorescence intensity profile shown in the graph to the right of image. Y axis represents fluorescence intensity, X axis represents the distance of white arrows. Chloroplast autofluorescence is shown in yellow. GFP channel was not applied to visualize hypha. H=Haustorium. Scale bar represents 10 μm.
