# Supplementary material for: The Phytophthora infestans Haustorium Is a Site for Secretion of Diverse Classes of Infection-Associated Proteins
Source: mBio. 2018 Aug 28;9(4):e01216-18. doi: 10.1128/mBio.01216-18 (PMC6113627; doi:10.1128/mBio.01216-18)
Supplement: TABLE S2 [file mbo004184040st2.docx]

**Table S2. Oligonucleotide primers for qRT-PCR and constructs for *P. infestans* transformation.**

| **Primer name Sequence (5’ – 3’)** |
| --- |
| qRT-INF4f CTCCACGTGCTCCAAGGATT  qRT-INF4r GCACATGGCCTTCTTCTCCT  qRT-01029f GGAGTGGCCAATCTTCCCAA  qRT-01029r AAGTCCCCGGCATCAAGAAG  Pi01029GWf AAAGCAGGCTTCACCATGCAGATATTTGCTCCCCT  INF4GWf AAAGCAGGCTTCACCATGAACTTCGTTGCCCTG  Pi22926GWf AAAGCAGGCTTCACCATGAAGCCTCTCCCGTTAGCA  attB-F2 GGGGACAAGTTTGTACAAAAAAGCAGGCT  attB-R2 GGGGACCACTTTGTACAAGAAAGCTGGG  mRFPgateway-R GAAAGCTGGGTCTTAGGCGCCGGTGGAGTG  Ham34KpnIf GGAAGGTACCGGGCCCATTATACC  Hmp1KpnIr GGAAGGTACCACCGTCGACCTCGA  PITG_21410 NotIf GGAAGCGGCCGCACCATGAACTTCGTTGCCCTG  PITG_21410 NotIr GGAAGCGGCCGCGCAAGCCTCTTACAGTCAGA  PITG_01029NotIf GGAAGCGGCCGCACCATGCAGATATTTGCTCC  PITG_01029NotIr GGAAGCGGCCGCGCCAGGAACGACTTGTCCAC  Pi22926 NotIf GGAAGCGGCCGC ACCATGCTCCGGTCCTTCTTACTr  Pi22926 NotIr GGAAGCGGCCGCGCTGTGGTAAGCTTCGTAAAACC |

Red font indicates restriction enzyme recognition sites: KpnI GGTACC, NotI GCGGCCGC
